# Study protocol for a stepped-wedge implementation study investigating the intersectoral collaboration of implementing the TREAT INTERACT intervention for primary school teachers and the mhGAP for health care workers for child mental health promotion in Uganda

**DOI:** 10.1186/s13063-024-08312-5

**Published:** 2024-07-09

**Authors:** Ane-Marthe Solheim Skar, Ingunn Marie S. Engebretsen, Nora Braathu, Harriet Aber, Harald Bækkelund, Melf-Jakob Kühl, Marjorie Mukisa, Joyce Sserunjogi Nalugya, Norbert Skokauskas, Vilde Skylstad, Tore Wentzel-Larsen, TREAT research consortium, Juliet Ndimwibo Babirye

**Affiliations:** 1 Norwegian Center for Violence and Traumatic Stress Studies, Gullhaugveien 1, Oslo, 0484 Norway; 2 Global Health Cluster, Division for Health Services, the Norwegian Institute of Public Health, Oslo, Norway; 3 Department of Global Public Health and Primary Care, Centre for International Health, University of Bergen, Bergen, Norway; 4 School of Public Health, Makerere University College of Health Sciences, MakSPH, Kampala, Uganda; 5 Regional Centre for Child and Youth Mental Health and Child Welfare, Faculty of Medicine and Health Sciences, Norwegian University of Science and Technology, Trondheim, Norway

**Keywords:** Adolescents, Children, Global mental health, Implementation, Low- and middle-income countries, Primary schools, Task-sharing, Teachers, Uganda

## Abstract

**Background:**

Mental and neuropsychological disorders make up approximately 14% of the total health burden globally, with 80% of the affected living in low- and middle-income countries (LMICs) of whom 90% cannot access mental health services. The main objective of the TREAT INTERACT study is to adapt, implement, and evaluate the impact of a novel, intersectoral approach to prevent, identify, refer, and treat mental health problems in children through a user centred task-sharing implementation of the TREAT INTERACT intervention, inspired by the World Health Organization (WHO) Mental Health Gap Action Programme Intervention Guide (mhGAP-IG) for primary school staff in Mbale, Uganda. Health care personell will be trained in the mhGAP-IG.

**Methods:**

This is a pragmatic mixed-methods hybrid Type II implementation-effectiveness study utilizing a co-design approach. The main study utilize a stepped-wedged trial design with six starting sequences, randomizing three schools to the intervention at each interval, while the remaining act as “controls”. Other designs include a nested prospective cohort study, case control studies, cross-sectional studies, and qualitative research. Main participants’ outcomes include teachers’ mental health literacy, stigma, and violence towards the school children. Implementation outcomes include detection, reach, sustainability, and service delivery. Child and caregiver outcomes include mental health, mental health literacy, and help-seeking behaviour.

**Discussion:**

Based on the results, we will develop sustainable and scalable implementation advice on mental health promotion and draft implementation guidelines in line with current WHO guidelines. This project will generate new knowledge on the structure, organization, delivery, and costs of mental health services in a LMIC setting, as well as new knowledge on the implementation and delivery of new health services.

**Trial registration:**

ClinicalTrials, NCT06275672, 28.12.2023, retrospectively registered.

**Supplementary Information:**

The online version contains supplementary material available at 10.1186/s13063-024-08312-5.

## Background

Mental health challenges present a high global burden, with stigma, inequity, poverty, and low prioritization of mental health care at a health systems level contributing to a relatively higher burden in low- and middle-income settings [1]. More than 80% of people with a mental health disorder live in low- and middle-income countries (LMICs). In Uganda, only 1% of the total health expenditure is used for mental health, including child and adolescent mental health (CAMH) [2]. Furthermore, there is a general lack of services and access to evidence-informed services, and it takes, on average, 17 years for only 14% of all research to reach the practice field [3]. Implementation research, “the scientific study of methods to promote the systematic uptake of research findings and other evidence-based practices (EBPs) into routine practice”, can help close this gap with particular relevance for LMICs [4, 5] when working towards universal health coverage [6]. Building on implementation science, mental health system strengthening and intersectoral collaboration between the health and other sectors [7], such as education, has the potential to increase both coverage and effectiveness of mental health care [8, 9]. In line with recommendations in the implementation science field, engaging key stakeholders and service users in mental health interventions is critical to empowering the workforce, enhancing mental health literacy, and combating stigma [8, 10, 11].

Education is a powerful tool to increase mental health literacy and break the stigma associated with mental health. Also, the school provides an opportunity for reaching children in need of services and following children over time [12]. Good child and adolescent mental health in countries with low-resourced health systems depends on the integration of health and education [13]. Healthy school environments are assumed to be related to improved education outcomes, and as such, there is an interdependence between good education and good child and adolescent development [14]. A systematic review of mental health promotion interventions in LMICs identified 22 studies during a 13-year period (2000–2013), including 14 school-based studies indicated that interventions aimed at promoting child and adolescent mental well-being can be implemented in school and community settings in LMICs with a positive impact on mental health outcomes [15]. A more recent systematic review, published in 2021, of universal school-based programmes focusing on anxiety and/or depression among children aged 6–18 years in LMICs identified six studies, most of which had clear methodological weaknesses [16] pointing towards a knowledge gap. Family and parenting interventions have also been employed to improve child and adolescent mental health (CAMH) in LMICs; however, a systematic review from 2019 revealed merely 36 papers on the topic [17]. Although research on services is scarce, we acknowledge that reviews may not fully capture the “reality on the ground” because the so-called “grey” literature or unpublished material is excluded. Yet, there is a clear scarcity of high-quality studies on CAMH and school-based interventions to promote child and adolescent mental health in LMIC primary schools.

Uganda has the highest proportion of children in the world, with nearly half of the population (47% or approximately 20 million) below the age of 15 [18]. A systematic review and meta-analysis including 26 papers found a prevalence of any mental disorder in Uganda of 22.9% in children, with anxiety disorders (14.4%) and depressive disorders (22.2%) being the most common [19]. Furthermore, many children are exposed to violence and trauma, which are important determinants of mental health problems [20]. In a study with 5804 children and adolescents aged 13–17 years in Uganda, 25% of girls and 11% of boys reported sexual abuse, 59% of boys and 44% of girls reported physical violence, and 20% of both girls and boys reported emotional violence [21]. Many children in Uganda also experience violence at school. In a representative sample of 3706 primary school children, 55% reported violence by their teacher [22]. However, fewer than 10% of children and adolescents who experience sexual abuse or physical violence receive help from health care services despite scoring significantly higher on mental distress than the non-exposed [21]. Child and adolescent mental health has been recognized as a serious public health and development issue by the Ugandan Government since the development of CAMH policy guidelines in 2017 [23]. Yet, in a scoping review including studies on CAMH services in Uganda [24], we described a limited set of mental health interventions launched between 2009 and 2019.

As in most sub-Saharan countries, the critical barriers to mental health provision in Uganda are a lack of resources and systems for referrals, stigmatizing attitudes, and a lack of help-seeking [25]. As of 2022, there were about 50 practicing psychiatrists in Uganda [26]. In 2017, only seven were trained in child and adolescent psychiatry, serving the country’s 20 million children [24]. Specialized clinical officers and nurses with shorter training primarily provide existing services. Task-sharing or task-shifting is practiced mainly by specialized and less specialized workers (e.g., with shorter training and fewer health care qualifications) [27, 28]. This results in work overload, inadequate training and supervision, and non-compliance with relevant guidelines [29].

As one of the strategies to build capacity and improve access to care, the Ugandan CAMH policy guidelines suggest “to establish mental, neurological and substance use services in schools and other institutions where children and adolescents are cared for.” While a school health workforce exists in Uganda, it fulfills in particular preventive measures in terms of vaccination, hygiene, and sporadic curative tasks. It runs ad hoc health information campaigns and similar tasks related to hygiene and somatic and reproductive health, while mental health remains largely neglected in such initiatives. Similar to the Ugandan health system, the education sector is under-resourced and faces implementation challenges at scale. The mainstream health care system provides access to children up to 5 years, subsequently there is a loss of contact during primary school age. The primary school setting has the potential to be an important arena for task-sharing interventions, with an enrolment rate of 98%, even if only 61% of children completed primary school in 2017 and only 32% completed without drop-out or repetition. Programmes that seek to change the behaviour of students and/or the current organization of schools (including timetabling, infrastructure, policies, and practices) require more intensive integration and collaboration than those which include only an educational or awareness-raising component [30]. Research is needed to understand how task-shifted mental health interventions can be implemented and sustained over time [31], their *acceptability and feasibility* among the workforce [28], their readiness to task shift, and how task-sharing can effectively address the mental health treatment gap.

The Mental Health Gap Action Programme Intervention Guide (mhGAP-IG) was first launched by the World Health Organization (WHO) in 2010 [32] and updated as mhGAP-IG Version 2.0 in 2016 [33]. This version revised the Child and Adolescent Mental Health Disorders model, which now covers developmental, behavioral, and emotional disorders among the target population, including middle childhood (6–12 year olds). It has a context-oriented approach, including general health care, carer, school, and the larger community. The mhGAP intervention aims to bridge the mental health treatment gap in countries with limited resources to achieve universal health coverage. Between 2012 and 2016, World Vision Uganda, together with the WHO and Uganda Ministry of Health, implemented a pilot mhGAP-IG project in three districts (Jinja, Kamuli, and Kitgum) in Eastern and Northern Uganda [34]. In 2016, a district mental health care plan was developed, and its integration into primary care was evaluated as “feasible” in the Kamuli district in Uganda [35]. These initiatives were predominantly focused on adult mental health, and mhGAP-IG has not been scaled up to other districts. Our study group has previously tested the applicability of the CAMH component of the mhGAP-IG in Uganda [36]. We found that the knowledge gain was equally high for nurses and clinical officers following 5 days of training, with initial but not prolonged changes in their practice. We believe this was due to a lack of focus at the system level on how this programme should be implemented sustainably, and concluded that supervision and “further task-sharing studies integrating CAMH into a larger sample of primary health care clinics are suggested, including a community mobilization component in the intervention to improve CAMH clinic attendance” [36].

In general, referral of children with mental health conditions to the public health system is very low in Uganda. This is partly due to a lack of available services and to stigma related to mental health problems [25] both at the health care system level and among community members [37, 38]. There is limited integration of mental health services into the existing health services and schools, and merely sporadic communication between services and sectors. Due to schools’ lack of operative resources, collaboration with other sectors has received little attention. However, a few intersectoral programmes have been scaled up and evaluated as effective [14, 28]. In Uganda, and to our knowledge in sub-Saharan Africa, CAMH is yet to be included in the tasks shared with the shifted education sector.

### Objectives

The overall aim of the TREAT INTERACT study is to adapt, implement, and evaluate the impact of the education and health interactive TREAT INTERACT intervention to prevent, identify, refer, and treat mental health challenges in children and adolescents in Uganda through a user-involved co-design approach. Our specific objectives are related to improving children’s mental health while simultaneously strengthening the education system’s handling and response of children with challenges as well as the intersectoral collaboration between health and education.

## Methods

### Trial design

**Fig. 1:**
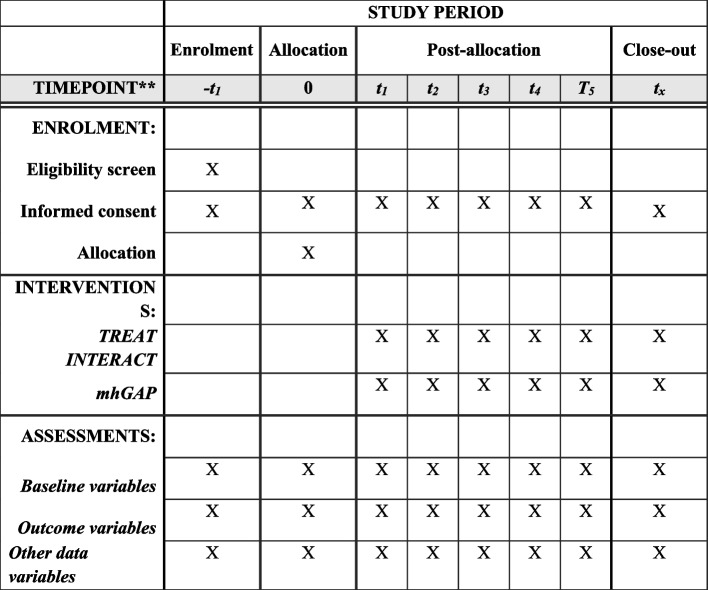
SPIRIT schedule of enrolment, interventions, and assessments. **See Supplementary material Tables 1 and 2

TREAT INTERACT will use a co-design approach where the research group and key stakeholders will collaborate at all stages of the research process according to implementation science theory and participatory action research. The study is designed as a pragmatic mixed-methods, Hybrid Type II Implementation-Effectiveness study [39], including dual testing of intervention effectiveness and evaluation of the implementation strategy using multiple study components. The SPIRIT reporting guidelines are adhered to [40] (see also Fig. 1).

Implementation science often relies on frameworks because of practical flexibility to facilitate implementation efforts [41]. The research will be directed by the consolidated framework for implementation research (CFIR) [42]. CFIR suggests consistent terms and definitions to make them applicable and enhance the generalizability of findings across settings, within and outside health care. In addition, the project will be organized within the Exploration, Preparation, Implementation, and Sustainment (EPIS) framework [43]. EPIS will be used as a conceptual tool to organize the various phases in the implementation process and different interrelated inner and outer factors likely to influence the implementation. Proctor’s [44] Implementation (acceptability, adoption, appropriateness, costs, feasibility, fidelity, penetration (reach), and sustainability), Service (efficiency, safety, effectiveness, equity, patient-centeredness, timeliness), and Client (satisfaction, function, symptomatology) Outcomes will guide the evaluation. We will strive towards making the intervention *gender-*sensitive, thus challenging potential socially constructed gender roles that might impact mental health in general, and when targeting gender-based violence in particular [45]. The intervention will also be *trauma*-sensitive by incorporating trauma and responses to trauma-exposure.

### Patient and public involvement

The research proposal was developed based on the TREAT consortium’s previous research within the same context. Key stakeholders were involved immediately after receiving funding to inform the development of the TREAT INTERACT intervention, and implementation strategies. The key stakeholder group were not involved in choice of outcomes measures or recruitment; however, they will be actively involved throughout all steps of the project.

### Study setting

The study will take place in Mbale District in Eastern Uganda, which was recently split into two administrative units: the District and the City. Mbale is located approximately 250 km northeast of Kampala. It has a population of approximately 500,000 people, half younger than 15 years. Eighty-six percent of those aged between 6 and 12 years attend primary school. Nearly all (97%) of the population live within 5 km of a primary school, and 94% live within 5 km of a public health facility.

Health care services are provided at five different levels: Level 1. Village health teams, Levels 2–4. Health centres II, III, IV, Level 5. Hospitals, and a regional referral hospital with a mental health unit. In Uganda, the administration of Districts is further divided into sub-districts, consisting of parishes. Villages are the smallest administrative unit. We will include a sufficient number of “sub-districts” that include primary health clinics (levels II, III), their respective hospital for supervision and referral (Mbal(e Regional Referral Hospital), and the relevant adjacent schools.

Within the health system, this study will target health workers at level III health centres who will be trained in the prevention and early identification of mental health problems, while those from health centre IV and hospital levels will receive extra training through the TREAT INTERACT in psychological and pharmacological treatment of childhood mental disorders and how to provide support and supervision to the teachers. At the district level, we will work with stakeholders in education, health, administration, and the police as well as the policy-makers to ensure sustainability in the referral system.

### Data collection and management

Data collection will be done on electronic devices by trained data collectors using the Open Data Kit (ODK) system and uploaded and stored on a Makerere University-based server. All data entered into the trial database via ODK will be subject to validation (number, date ranges, etc.) to assure the quality of the resulting dataset. Important trial documents and databases will be archived for at least three years after completion of the clinical trial. Data collectors will thoroughly review the consent form with teachers at the school prior to data collection. Data collectors review the consent and assent form individually with caregivers and children as well and specify what type of questions will be asked. Throughout data collection, it is made very clear that participants can choose to withdraw at any time or not answer every question.

Qualitative data will be stored and backed up in predefined data management systems within Makerere. A data-sharing contract is elaborated between the institutions. The statisticians, PIs, and PhD students will serve as a data monitoring committee and have access to the interim results to monitor the data collection and the final dataset. Midway in the data collection, the same team will be auditing the trial process, including enrolment, consent, intervention adherence, and data completeness. This trial does not involve collecting biological specimens for storage.

### Work packages

This project is divided into four work packages (WPs). WP2 is the largest in terms of allocated resources and data collection efforts and comprises a stepped-wedge randomized controlled design. The work packages are:

- WP1: Intervention and implementation mapping
- WP2: School implementation
- WP3: Intersectoral collaboration
- WP4: Sustainability

#### WP1: intervention and implementation mapping

The main objective of this work package was to develop a school intervention, the TREAT INTERACT intervention, inspired by the mhGAP-IG and develop implementation strategies among the stakeholders in primary schools in Mbale to inform the policy formulation and implementation of mhGAP in the health centres and the TREAT INTERACT in the schools to ensure that the education and health system has similar understanding of child mental health promotion and treatment. An implementation map is made to document the process from the initial exploratory phase until evaluation and dissemination. Data will be collected using mixed methods, and a theory of change will be created through a participatory approach with user involvement. Interviews will be conducted in English and transcribed verbatim. For our project, we have started by mapping stakeholders in the field of child and adolescent mental health (child and adolescent specialists from different sectors, police, and policy professionals). Consultative meetings are held with these stakeholders to guide the development of the TREAT INTERACT intervention and to assess its suitability for the planned trainings.

Together with these stakeholders, a root cause analysis is done to establish the root causes of the current situation of mental health service delivery for children and adolescents. Through this participatory approach with stakeholders in child and adolescent mental health, we aim to set the ground for participation, ownership, and collaboration between the various sectors for the improvement of referral systems and mental health outcomes among school-going children [46].

#### WP2: school implementation

In this work package, the project will estimate the effect of the TREAT INTERACTION school intervention among teachers on implementation and client outcomes in primary schools in Mbale to inform the implementation of child and adolescent mental health services. Specifically, we shall measure the readiness and level of reach of child and adolescent mental health services in Mbale. We shall also determine which implementation strategies are associated with the sustainability of the TREAT INTERACT school training programme in primary schools and the health sector in Mbale.

The quantitative aspects in this work package will be a stepped-wedge cluster randomized trial to randomize 18 schools into six cohorts of three schools. Using the stepped-wedge design will allow every participant to receive the intervention. All cohorts will contribute data throughout 21 months, during both the control and intervention phases. All cohorts will provide data at baseline before any of the schools receive the intervention, at month 0, and then repeatedly every three months adjusted for school terms. Cohort 1 will receive the intervention at month 0; cohort 2 at month 3, cohort 3 at month 6, cohort 4 at month 9, cohort 5 at month 12, and cohort 6 at month 15.

The primary outcomes in this work package will be the effect of task-sharing using the TREAT INTERACT school programme on stigma, mental health literacy, and violence. The 18 schools are systematically selected using STATA and according to two strata: urban or rural location and public or privately owned schools. All teachers will be enrolled for study participation. This will be an open-cohort study where new employees, starting employment after the intervention has been rolled out, will be instructed to complete an information meeting within 4 weeks following their employment, and then take part in the remainder of the data collection. New training courses will be provided to new entrants when needed.

##### Eligibility criteria

Participants eligible for the trial must comply with any of the following at randomization: A teacher/ staff member at a preselected primary school in Mbale. Child-caregiver pairs are eligible when a learner is enrolled in a selected primary school in Mbale, the child has a caregiver living with her or him and provides ascent, and the caregiver with a child in the selected school provides informed consent.

##### Sample size

Power analyses were conducted in R [47] using the calcPower.SWD function in the samplingDataCRT package and indicated acceptable power, at least 80%, for realistic effect sizes in a stepped wedge design with cluster (school) as a random effect. Longitudinal inclusion of teachers is assumed since it is assumed that most teachers will stay in the schools during follow-up. This model assumes that the within-cluster variance, between-cluster variance and the error variance are equal. Based on the expected sample size of 10 teachers per school, we can expect to detect a treatment effect of 0.2, which is considered a small effect size. However, since preliminary analysis of the situation showed fewer teachers at most schools, a decrease that became apparent during Covid-19, all teachers in the sampled schools will be included in the study.

For the child-caregiver pairs, a sample size of 388 was found sufficient to detect a proportion difference of 0.50 compared to 0.30 with 80% power and 10% loss to follow-up [48]. A stratified sampling proportional to the student population size in each school is used to determine the number of child-caretaker pairs to be included per school. To measure the impact of the intervention among teachers on the community, child-caretaker pairs will be enrolled in a mixed methods design with a nested prospective cohort study to collect quantitative data and conduct focus group discussions and key informant interviews. For the nested prospective cohort, individual study units (child-caretaker pairs) will be selected using systematic sampling. We shall obtain the registration lists of each cohort from the selected primary schools in Mbale. The sampling will start by selecting an element from the list at random (random starting point = P) using computer-generated random numbers. Every K^th^ element in the frame will be selected, where K is the sampling interval. The individuals selected for study enrolment will be done as follows:

- First respondent will have the number = P, 2nd respondent will have the number = P + K, 3rd respondent will have the number = P + K + K. The selection process will be continued until the number of study units (388 child-parent pairs) required per school is attained. At the same time, sex and age-stratified focus group discussions and stakeholder key informant interviews will be employed to collect qualitative data. The mixing of methods aims to establish corroboration of data and will be a concurrent triangulation design.

##### Randomization

Schools will be identified through a list of schools in the target area, including information on number of learners and teachers, and urban and rural localization. A randomization procedure for school inclusion will be made. Following this, project group members and trained recruiters will recruit the schools. The schools will be sorted into six cohorts randomly incrementally included within 18 months. This design provides within-unit data from the cohorts when they are in a control and intervention condition, and between-unit data between the cohorts in the control versus intervention condition. The procedure used for randomization is stated in detail in an R function. There is no risk of bias when using the R function. Since the number of schools is fixed at 18 there is no need for blocking. As this is cluster randomization, there is no possibility of blinding, and therefore neither concealing.

##### Intervention

The Ugandan-adapted CAMH TREAT INTERACT intervention, inspired by the mhGAP-IG, will be used for the identification, assessment, and management of common mental disorders in children and adolescents whereas the healthcare workers will be trained in the original mhGAP-IG [49]. The clinical decision-tree guides the assessment and differential diagnostic process towards identifying developmental problems (delayed or disrupted development), problems with inattention or hyperactivity (ADHD), conduct disorder (abnormally aggressive, disobedient or defiant behaviour), emotional problems (severe distress, sadness, fearfulness, anxiety or irritability). Examples of typical presentations across different ages and developmental stages are provided to assist the evaluation. Clinicians are also instructed to assess somatic symptoms, home-environment, and school-environment to ensure a comprehensive evaluation. The intervention further details six different protocols for the management of these mental health problems, primarily based on psychosocial and systemic interventions. The described psychosocial interventions can also be provided as general prevention for children with subclinical problems. Lastly, the module guides further follow-up assessment. Experts identified by the Ministry of Health will train trainers who train and follow up teachers and health personnel receiving the intervention.

##### Adherence

To ensure adherence to the intervention, supportive supervision will be given every month for the first three months, then every quarter for the rest of the study period. There will be refresher training every 3rd month after implementation, in line with Ministry of Health guidelines.

##### Outcomes

To be able to compare the two conditions (intervention vs. wait-list), the same variables will be measured before randomization, as well as during and following enrollment to the intervention. To investigate potential gender differences, aggregated information will be separated by gender.

##### Quantitative measures to be collected by each participant group

A detailed supplementary table lists the relevant concepts and chosen scales for documenting the implementation (Supplementary Table 1). In short, we collect data on the school level, teacher and health care level, and child-caretaker level.

##### School level

Children receiving identification, counselling and/or referral for mental health conditions at school: number of home contacts; number of counsellings; and number of referrals. Data will be collected by the appointed mental health focal point teachers at each school and registered into a safely stored book. Data will be registered continuously.

##### Teacher and healthcare level

Data will be collected by trained research assistants every three months. Research assistants will visit each school to collect the data. The following data will be collected:

- Measures about the school children: Stigma of child mental health, knowledge about mental health (literacy), personal mental health, attitudes about gender norms, access to suitable MNS services, and use of violence towards school children.
- Service measures: Access to MNS health services, users treated for MNS disorders, availability of psychosocial interventions, and use of MNS services.
- Implementation measures: Programme sustainability, leadership, organizational readiness, acceptability, feasibility, fidelity.
- Reach (scale developed for the current study): Reach refers to the number of children receiving mental health services at each school and the number of referrals and services in health facilities.

Data from healthcare workers will be collected qualitatively after implementation starts.

##### Caregiver level

Data will be collected by trained research assistants every three months. Research assistants will visit each school to collect data individually from each caregiver who agrees to participate. The following data will be collected: Stigma of child mental health, help-seeking behaviour, knowledge about mental health (literacy), discipline, alcohol, and substance use.

##### Child level

Data will be collected by trained research assistants every three months. Research assistants will visit each school to collect data individually from each child whose caregiver has agreed to participate. The following data will be collected: Child mental health, support from teachers, alcohol use, teacher violence, treatment at home (discipline).

##### Data analysis plan

Mean values will be used as summary measures, and mixed effects models will be used to examine the relationships of outcomes with intervention variables and secondarily with other background variables. Background variables will be measured before randomization. Changes between time points are of interest, but changes between the pre- and post-intervention are of particular interest in addition to differences between control and intervention clusters. Based on the repeated measure data, mixed effects analysis will be conducted for outcome variables at the teacher and child level. Mixed effects models are fit to handle any missing data that may occur during data collection under the less restrictive assumption Missing at random. We will use NVivo software for qualitative data analysis when appropriate.

##### Retention

To mitigate both non-retention and non-adherence, once the teachers are enrolled or randomized, the study site will make an effort to follow them for the entire study period. It is projected that the rate of loss-to-follow-up on an annual basis will be at most 5%. Study site staff will develop and implement local standard operating procedures to achieve this level of follow-up.

#### WP3: intersectoral collaboration

##### Primary objective

To implement and evaluate the mhGAP-IG in the health sector while also investigating intersectoral supervision, communication, and referral model between the health and education sector.

##### Specific objectives

(i) To measure the effect of the mhGAP-IG training programme on stigma, mental health literacy, diagnosis, and treatment among health workers in Mbale.
(ii) To develop and implement an intersectoral supervision, communication, and referral model between Mbale´s education and health sectors, where teachers have been trained in TREAT INTERACT and the health care providers in the mhGAP-IG.

##### Design

Data will be collected parallel to the data collection in WP2 (baseline, 6 data collections, follow-up) (Supplementary Table 2).

##### Participants

Participants include class *teachers and school heads* from WP2 and trained personnel at clinic level III (there are four clinic levels before hospital level, of which level III can be found in every sub-county), sub-district level and hospitals. The *health clinic personnel* regularly interacting with schools are usually nurses and clinical officers (3–4 years of clinical medical training) who sporadically visit schools for ad-hoc health campaigns. In addition, caregivers and children from the participating schools will provide data.

##### Procedure

**Fig. 2:**
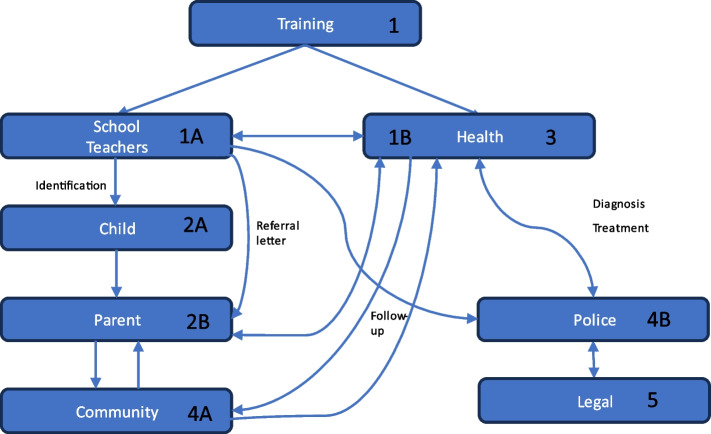
Child mental health care pathway

This WP has two training and implementation interventions. First, we plan to train all permanent primary health clinics, level III clinical staff (nurses and clinical officers) in the 6 clinics representing 6 sub districts in 2–3 rounds until all permanent staff having clinical counselling tasks with children are reached (nurses and clinical officers), followed by training of their referral hospital affiliated supervisors in the use of mhGAP-IG. We plan to use a 5-day mhGAP intensive training course that has proven efficient in knowledge gain [50]. The health personnel provided for by the Uganda Mental Health Act of 2019 will be trained to deliver the TREAT INTERACT training at the schools (training of trainers), thus initiating a personal link with schools. Secondly, trained as trainers and equipped with simple materials, the TREAT INTERACT and mhGAP-IG trainers (nurses and clinical officers) will provide the TREAT INTERACT intervention comprising lectures, discussions, and role-play followed by homework and follow-up activities to class teachers, providing an easy guide how to detect children with common mental health challenges (delivery modes and content is determined in WP1). School heads and community leaders will participate in the training, which is in line with the World Bank Group Strategy for Fragility, Conflict, and Violence (2020–2025) [51]. This orientation of teachers and school heads will also include referral instructions. The child mental health care pathway is shown in Fig. 2.

As demonstrated in Fig. 2, each new number illustrates an action step in the TREAT INTERACT referral structure. The action may be judged sufficient at each referral level (represented as a box). Interactions between the school and health sector about a child can only happen with consenting parents unless in cases of emergency. The involvement of a select group of community members is to provide support in implementing interventions such as psychological first aid alongside the teachers [4a]. Both teachers and health workers may contact the police without parental consent according to ethical obligations [4b&5].

Once a child with severe difficulties is identified by the teacher (1), the school head will initiate the steps to follow (2). Caregivers should be involved at this stage, unless the child may face harm. If so, an adult carer (approved by the official village administrative structures) will be identified. The key intersectoral step involves the contact nurse or clinical officer as part of school referral to the primary health sector (3). The primary health sector decides on the need for further referral to more specialized mental health services. A child-centred plan for the child in school should be agreed upon based on symptoms and function findings, involving the school, health personnel, and carer(s). Some of these steps may be repeated, and a regular evaluation is included. Based on this work and insights from WP1, we will develop training guidelines for teachers and school heads. A simple referral system in collaboration with local stakeholders will be integrated into existing systems. Across health centres, a competent team of trainers will be established to integrate with the education sector on initial CAMH management.

##### Measures

Client outcomes (see WP2) will be completed by health personnel. Qualitative interviews (focus groups and key-informant interviews) will be conducted to collect data on client outcomes from health personnel. This data will be collected after start of implementation. Intersectoral collaboration will be measured through referrals, diagnosis, and treatment.

*Reach* and *service outcome* measures will be developed in WP1.

#### WP4: sustainability

##### Primary objective

To develop evidence-supported implementation strategies for the sustainability of CAMH services to inform policy formulation and implementation.

##### Specific objectives

(i) To describe the implementation factors, strategies, frequency of interaction, and sustainment of the intersectoral collaboration between health and education sectors.
(ii) To assess the process and associated costs of integrating the TREAT INTERACT and mhGAP interventions into Mbale’s education and health sectors.
(iii) To develop policy advice and incentive mechanisms to ensure functional and sustainable collaboration between educational and health sectors.

##### Participants

We will use a participatory action research user involvement approach (plan, act, observe, reflect; PAR) to strengthen intersectoral communication and collaboration and conduct qualitative interviews with local stakeholders and national decision-makers with a focus on sustainment. The study participants will be identified purposively and sampled using the snowball methodology. The key-informant interviews shall be conducted in English by two co-investigators and transcribed verbatim.

##### Methods

We will conduct framework analysis [52]. The approach to analysis by Gale will be adopted as a multi-disciplinary perspective as advised when exploring perspectives in multi-sectoral fields (education, health, local governance) at an administrative level between executive and operative levels [53].

##### Procedure

In this package, a PAR approach will be used. Key stakeholders that were included in WP1, as well as other relevant key stakeholders from the education and health sector and the Ministry of Health and the Ministry of Education, will be identified and invited to a workgroup to address implementation barriers and catalysts that need to be further addressed to secure sustained practices. A focus lies on incentive mechanisms and vertical performance management in the health and education sectors. Interviews with decision-makers will address institutional and procedural aspects for scaling up the task-sharing between sectors. Results from the intervention and implementation mapping (WP1) and follow-up data (WP2), as well as cost estimations (WP3), will be illustrated in templated materials for scalable implementation strategies and institutional stakeholder maps [54] at 3 levels: (1) management guidelines for school heads that instruct personnel in systematic integration of CAMH detection and referral, and (2) simple guidance material for teachers to obtain during mental health awareness training. (3) At the national level, we will develop stakeholder-informed guidelines for piloting and scaling up intersectoral collaboration. Combining all WPs, we will provide evidence-based strategies and advice on feasible collaboration to improve CAMH between both the education and health sectors at the management level, relying on existing resources and personnel. We will assess the impact in clinics based on special referral forms from the project.

## Discussion

### Dissemination

A multichannel approach will be used for study results dissemination, including peer-reviewed publications, conference presentations, reports, and media outreach. Policy briefs and advice will be made for policymakers. Visually engaging infographics will be developed to present key findings for easy comprehension concisely. Ongoing collaboration with stakeholders will inform the dissemination strategy.

### Strengths and limitations

Implementation research from LMIC is essential to reach universal health coverage [55]. This is the first implementation study of mhGAP-IG in a school setting, with the potential to be implemented across several LMICs. The active inclusion of key stakeholders from the health and education sector as well as religious leaders and the police enhances the relevance of the research and helps in developing sustainable implementation strategies. The CFIR has been optimized for use in LMIC settings [56], with the current study being among the first to use the updated consolidated framework for implementation research in an LMIC. Stepped wedge designs, similar to other cluster-randomized designs, excel in minimizing the potential for contamination. There is no contamination within schools, and remaining contamination is between schools. We will recommend teachers to avoid bringing instruction material out of schools. A challenge with stepped-wedge designs is however the long data collection period, and in the current study, the many data collection points, potentially affect the truthfulness of the responses due to fatigue.

### Expected impact and implications

The overall aim is to strengthen child and adolescent mental health, which can have broader impacts on the communities. Our suggested task-sharing approach may, if successful, be scaled up within Uganda and tested and scaled up in other LMICs. Further, task-sharing might also be relevant for higher-income countries where mental health facilities are not available for all, such as in the USA, where 70% of counties in 2016 did not have a child psychiatrist [57]. Although the mhGAP is widely used and studied across LMICs, few studies have used a randomized design, and no study has, to our knowledge, tested the mhGAP as an integrated CAMH approach between the health and education sectors. This study will also develop comprehensive knowledge related to user involvement in strengthening mental health services in LMICs [8, 58].

Uganda has made progress related to poverty reduction and life expectancy. However, a weak focus on mental health resulted in guidelines that have not been implemented, leaving children in need of services without any support. This study will investigate how evidence-supported mental health programmes can be implemented and sustained in a resource-constrained context by identifying effective implementation strategies to ensure sustainability and provide comprehensive data on the effect of delivering a well-recognized, simplified mental health programme (mhGAP), and the newly developed sister intervention (TREAT INTERACT) as part of a task-sharing approach, highlighted as vital to make interventions available and to reach global mental health coverage in LMICs [59, 60].

## Trial status

Protocol version number: 1, date: 26.3.3034. Trial registration: ClinicalTrials NCT06275672. Registered 28.12.2023, NCT06275672. Recruitment started September 2023, and data will be collected until June 2025. Trial registration was delayed due to logistic and technical problems. After registration in ClinicalTrials there are a total of six additional data collections.

## Supplementary Information

Supplementary Material 1: Supplementary Table 1. Concepts with chosen outcomes, outcome measures and planned analysis.

Supplementary Material 2: Overview of when each cohort will provide data and what data is collected, and when each cohort will receive the intervention.

Supplementary Material 3: Reporting checklist for protocol of a clinical trial (SPIRIT guidelines).

## Data Availability

Technical appendix, statistical code, and dataset will be provided upon reasonable request.

## Abbreviations

LMIC: Low- and middle-income countries
CAMH: Child and adolescent mental health
mhGAP-IG: Mental Health Gap Action Programme—Intervention Guide
EBPs: Evidence-Based Practices
SDG: Sustainable Development Goals
WHO: World Health Organization
ODK: Open Data Kit
