# Supplementary material for: Study protocol for a stepped-wedge implementation study investigating the intersectoral collaboration of implementing the TREAT INTERACT intervention for primary school teachers and the mhGAP for health care workers for child mental health promotion in Uganda
Source: Trials. 2024 Jul 9;25:465. doi: 10.1186/s13063-024-08312-5 (PMC11234609; doi:10.1186/s13063-024-08312-5)
Supplement: Supplementary file 1 — Supplementary Material 1: Supplementary Table 1. Concepts with chosen outcomes, outcome measures and planned analysis. [file 13063_2024_8312_MOESM1_ESM.docx]

Supplementary Table 1: Concepts with chosen outcomes, outcome measures and planned analysis

| **Primary outcomes** | | | | |
| --- | --- | --- | --- | --- |
| Variable/outcome | Level | Hypothesis / research questions | Outcome Measure(s) | Methods of Analysis |
| 1.Implementation measures |  |  |  |  |
| a. Reach | Teachers | Cohorts included at earlier time points will have a higher mental health detection rate and higher number of referrals to the mental health system than cohorts included at later time points. There will be higher reach post-intervention compared to pre-intervention. | - Number of teachers participating in the program - Proportion of children reached by the program | - Descriptive statistical analyses will be conducted to analyze the reach data. The total number of participants will be summarized, and proportions will be calculated to determine the percentage of the target population reached. |
| b. Sustainability | Teachers | The implementation of the TREAT INTERACT and intervention will contribute to the long-term sustainability of mental health support and services for children and adolescents at primary schools | - Financial Stability - Organizational Support - Staff Retention - Program Integration - Stakeholder Perceptions - Program Outcomes and Impact | - Sustainability tool (NoMAD, Finch et al., 2013, and Provided Report of Sustainment Scale, Moullin et al., 2021) - Qualitative: participatory action research with user involvement. |
| 2. Service measures |  |  |  |  |
| a. Access to mental health care | Health personnel | There will be an improvement in the access to mental health services.  Implementation of the TREAT INTERACT and mhGAP interventions will result in improved access to mental health care services for children and adolescents | - Service Utilization - Wait Times - Geographical Accessibility - Affordability - Equity and Disparities - Satisfaction and Perceived Access - Referral Patterns - Availability of Services | - Number of individuals who access mental health care services within a specified period - Assess the average wait time for individuals seeking mental health care services. - Evaluate the distance and travel time individuals need to access mental health care facilities - Determine the affordability of mental health care services by assessing the out-of-pocket costs, insurance coverage, or availability of subsidies and financial assistance programs |
| 3. Client measures |  |  |  |  |
| a. Teacher attitudes and knowledge about child mental health | Teachers, health personnel and caregivers | There will be a decrease in stigma and increase in knowledge following the implementation of the TREAT INTERACT school program | - Attitudes about Child Mental Health (Perceived Discrimination-Devaluation, Link et al., 2004) - Stigma Reduction - Mental Health Literacy (MAKS: Evans-Lacko et al., 2010) - Client Satisfaction | - Mixed effects models: changes in knowledge and understanding of child mental health conditions - Assessments of their overall experience, perceived effectiveness of interventions, and satisfaction with the support and guidance provided by mental health professionals - Qualitative interview analysis |
| b. Violence | Teachers, health personnel, caregivers | There will be a decrease in violence following the implementation of the TREAT INTERACT school program | - The dimensions of discipline inventory (DDI: Strauss & Faucher, 2007). - Incidents of Teacher Violence - AUDIT alcohol use among caretakers, WHO - School Policies and Practices | - Mixed effect analysis - Qualitative interview analysis |
| **Secondary outcomes** | | | | |
| Variable/outcome |  | Hypothesis | Outcome Measure | Methods of Analysis |
| 1.Implementation measures | | | | |
| a. The perception of acceptability, appropriateness, feasibility, ownership, school climate and user participation | Teachers,  (only head teachers for ORIC) | Participants rating the intervention with high implementation scores will respond better to TREAT INTERACT (higher Reach, lower mental health stigma, and less teacher violence) | - The Implementation Quality Questionnaire (Bogen, 2020) - Organizational Readiness (ORIC, Shea et al., 2014) | - Mixed effects analysis for teacher and child level data - Qualitative analysis |
| b. Fidelity | Teachers | Participants with high fidelity to the intervention will report higher reach, lower mental health stigma, and less teacher violence | - Fidelity Scale - Adaptations and Modifications - Contextual Factors | - Qualitative analysis |
| 2.Client outcomes |  |  |  |  |
| a. Personal Mental Health | Teachers, health personnel | Participants with higher levels of mental health problems will have reduced symptoms after implementation of the TREAT INTERACT school program | - General Health Questionnaire (GHQ, Goldberg, 1970) | - Mixed effects analysis - Qualitative analysis |
| b. Gender norms | Teachers, health personnel | There will be a decrease in traditional gender beliefs after implementation of the TREAT INTERACT school program | - Attitudes on Gender Norms (Waszak et al., 2000) | - Mixed effects analysis - Qualitative analysis |
| c. Help-seeking | Caregivers | Caregivers who are more open to seek help for mental health issues will have less stigma and more knowledge on mental health | - Help-seeking behaviour (Stan Kutcher & Yifeng Wei, 2017) | - Descriptive and mixed effects analysis |
| Child outcomes | | | | |
| Variable/outcome | Level | Hypothesis | Outcome Measure | Methods of Analysis |
| Child mental health | Child | There will be a reduction of mental health symptoms after implementation of the TREAT INTERACT school program | - Pediatric Symptom Checklist (PSC-17: Jellinek et al., 1998) | - Mixed effects analysis - Qualitative analysis |
| Support from teachers | Child | Support from teachers will increase after implementation of the TREAT INTERACT school program | - Teacher Support Scale (TSS; Metheny, McWhirter, & O’Neil, 2008) | - Mixed effects analysis - Qualitative analysis |
| Teacher Violence | Child | Teacher violence will decrease after implementation of the TREAT INTERACT school program | - Teacher violence scale (Piskin et al., 2014) - Questions about sexual violence, self-made | - Mixed effects analysis - Qualitative analysis |
| Discipline at home | Child | Examining the child’s experiences at home and if it interferes with violence experiences at school | - Treatment at home - The dimensions of discipline inventory (DDI; Strauss & Faucher, 2007) | - Mixed effects analysis |
