# Supplementary material for: Study protocol for a stepped-wedge implementation study investigating the intersectoral collaboration of implementing the TREAT INTERACT intervention for primary school teachers and the mhGAP for health care workers for child mental health promotion in Uganda
Source: Trials. 2024 Jul 9;25:465. doi: 10.1186/s13063-024-08312-5 (PMC11234609; doi:10.1186/s13063-024-08312-5)
Supplement: Supplementary file 2 — Supplementary Material 2: Overview of when each cohort will provide data and what data is collected, and when each cohort will receive the intervention. [file 13063_2024_8312_MOESM2_ESM.docx]

Supplementary Table 2: Overview of when each cohort will provide data and what data is collected, and when each cohort will receive the intervention

|  | **M0** | **M1** | **M2** | **M3** | **M4** | **M5** | **M6** | **M7** | **M8** | **M9** | **M 10** | **M** **11** | **M** **12** | **M** **13** | **M** **14** | **M** **15** | **M** **16** | **M** **17** | **M** **18** | **M 19** | **M 20** | **M 21** |
| --- | --- | --- | --- | --- | --- | --- | --- | --- | --- | --- | --- | --- | --- | --- | --- | --- | --- | --- | --- | --- | --- | --- |
| C1 | BL+W1 |  | - | P1 |  |  | P2 |  |  | P3 |  |  | P4 |  |  | P5 |  |  | P6 |  |  | S |
| C2 | BL+W1 |  |  | W2 |  |  | P1 |  |  | P2 |  |  | P3 |  |  | P4 |  |  | P5 |  |  | S |
| C3 | BL+W1 |  |  | W2 |  |  | W3 |  |  | P1 |  |  | P2 |  |  | P3 |  |  | P4 |  |  | S |
| C4 | BL+W1 |  |  | W2 |  |  | W3 |  |  | W4 |  |  | P1 |  |  | P2 |  |  | P3 |  |  | S |
| C5 | BL+W1 |  |  | W2 |  |  | W3 |  |  | W4 |  |  | W5 |  |  | P1 |  |  | P2 |  |  | S |
| C6 | BL+W1 |  |  | W2 |  |  | W3 |  |  | W4 |  |  | W5 |  |  | W6 |  |  | P1 |  |  | S |
| **Assessments** | | | | | | | | | | | | | | | | | | | | | | |
| Baseline questionnaire variables: | | | | | | | | | | | | Demographic information (age, sex, level of education, etc.), referral of children, mental health stigma, mental health knowledge, gender norms, discipline behaviours, personal mental health, and implementation questions | | | | | | | | | | |
| Outcome variables collected at all time points: | | | | | | | | | | | | Referral of children, mental health stigma, mental health knowledge, discipline behaviours, personal mental health, and implementation questions | | | | | | | | | | |
| Other data variables: | | | | | | | | | | | | Headteachers are asked about organizational readiness after the implementation of the intervention | | | | | | | | | | |

*M = Month, C = cohorts, BL = baseline data, W = waitlist condition, P = post-intervention questionnaire, S = sustainability phase. Blue areas indicate when the cohort will receive the TREAT INTERACT intervention – directly after waitlist condition data collection.
